# P16 and Ki67 Immunostains Decrease Intra- and Interobserver Variability in the Diagnosis and Grading of Anal Intraepithelial Neoplasia (AIN)

**DOI:** 10.4137/cpath.s501

**Published:** 2008-02-09

**Authors:** Ann E. Walts, Juan Lechago, Bing Hu, MaryBeth Shwayder, Lynn Sandweiss, Shikha Bose

**Affiliations:** 1 Department of Pathology and Laboratory Medicine, Cedars-Sinai Medical Center, Los Angeles, CA; 2 Department of Pathology and Laboratory Medicine, UCLA Medical Center, Los Angeles, CA

**Keywords:** human papilloma virus (HPV), anal intraepithelial neoplasia (AIN), condyloma, P16, Ki67, intraobserver variability, interobserver variability

## Abstract

**Background:**

Significant variation is reported in the diagnosis of HPV-associated AIN. We previously observed that band-like positivity for p16 in >90% of contiguous cells coupled with Ki67 positivity in >50% of lesional cells is strongly associated with high grade AIN. This study was undertaken to determine if addition of p16 and Ki67 immunostaining would reduce inter- and intraobserver variability in diagnosis and grading of AIN.

**Design:**

H&E stained slides of 60 anal biopsies were reviewed by three pathologists and consensus diagnoses were achieved: 25 negative, 12 low (condyloma and/or AIN I) and 23 high (9 AIN II and 14 AIN III) grade lesions. The H&E stained slides were diagnosed independently by three additional (“participant”) pathologists. Several weeks later they re-examined these slides in conjunction with corresponding p16 and Ki67 immunostains.

**Results:**

Addition of p16 and Ki67 immunostains reduced intra- and interobserver variability, improved concurrence with consensus diagnoses and reduced two-step differences in diagnosis. Negative and high grade AIN diagnoses showed the most improvement in concurrence levels.

**Conclusion:**

Addition of p16 and Ki67 immunostains is helpful in the diagnosis and grading of AIN.

## Introduction

The incidence of anal squamous carcinoma and its precursor lesions has increased in recent years although the true incidence of anal intraepithelial neoplasia (AIN) is unknown (Shepherd, 2007). These lesions are most prevalent among HIV+ men who participate in anal receptive sex (MSM) (Daling et al. 1982; Melbye et al. 1994; Palefsky et al. 1998; Daling et al. 2004). Prior to the human immunodeficiency virus (HIV) epidemic the incidence of anal cancer in this high risk population was estimated at 36.9 per 100,000 (Daling et al. 1982), similar to the incidence of cervical cancer prior to the establishment of routine cervical cytology screening programs. In HIV positive individuals the incidence of anal cancer has been estimated to be twice that in HIV negative individuals (Melbye et al. 1994; Goedert et al. 1998). The American Cancer Society projected that in 2007 about 4650 new cases of anal cancer would be diagnosed in the United States (up from 3400 cases in 2000 and 4010 cases in 2004) and that about 690 persons (up from 580 in 2004) would die of the disease during the year (American Cancer Society). The increased incidence of AIN is most likely due to both an increase in HPV and HIV infections and the increase in longevity attributable to antiretroviral therapy which does not appear to be effective against HPV infections or HPV associated neoplasia (Frisch, Biggar, Goedert, 2000; Horster et al. 2003; Ortholan, Francois, Gerard, 2003; Palefsky et al. 2001).

As is the case for squamous lesions in the cervix, human papilloma viruses (HPV) have been shown to play an important role in the pathogenesis of the vast majority of these lesions (Palefsky, 2000). At present there exist significant differences in the interpretation of anal biopsies that impact patient management (Colquhoun et al. 2003; Haber et al. 2004; Lytwyn et al. 2005). Factors that contribute to intra- and interobserver variation in the interpretation of anal biopsies for intraepithelial neoplasia (AIN) include application of subjective criteria, tangential sectioning, small size of biopsies, coexistent reactive/inflammatory atypia and thermal artifact. In a recent study of anal biopsies (Walts, Lechago, Bose, 2006a), we found that (a) a band-like pattern of p16 immunostaining coupled with Ki67 positivity in >50% of the lesional cell nuclei was strongly associated with high grade AIN; (b) absence of a p16 band of immunoreactivity, coupled with Ki67 positivity in <50% of lesional nuclei, was frequently associated with benign lesions; (c) most AIN I lesions stained similar to the non-dysplastic cases; (d) band-like p16 positivity correlated strongly with the presence of high risk HPV DNA as determined by in-situ hybridization. The current study was undertaken to ascertain whether addition of p16 and Ki67 immunostains, interpreted in accordance with the findings listed above reduces intra- and interobserver variability in the histologic diagnosis and grading of AIN.

## Materials and Methods

After IRB approval, H&E stained sections of 60 formalin fixed anal tissue samples from 52 individuals (51 biopsies from 43 males and 9 biopsies from 9 females) were retrieved from the surgical pathology files of our department. Males ranged in age from 28 to 57 years (mean: 41 yrs; median: 40 yrs) and females ranged in age from 19 to 72 years (mean: 50 yrs; median: 48 yrs). Cases were selected to include an assortment of squamous lesions. No additional selection criteria were applied. HIV status was not known.

All slides were reviewed by two pathologists. After diagnostic differences had been resolved by discussion and/or review by a third pathologist, the following consensus diagnoses were achieved: 25 negative (reactive, inflammatory, and/or hemorrhoids), 12 low grade squamous intraepithelial lesions (condyloma and/or AIN I), and 23 high grade squamous intraepithelial lesions (9 AIN II and 14 AIN III). The slides, designated 1 to 60, were subsequently diagnosed independently, and without knowledge of prior diagnoses, by three additional pathologists who routinely sign out anal biopsies, herein designated participant pathologists. Diagnoses were recorded as negative, low grade AIN, or high grade AIN as explained above. Only one diagnosis was permitted per case. In sections showing combinations of HPV associated change and/or various grades of AIN, the highest grade lesion was recorded as the working diagnosis.

One month to six weeks later, during which time the three participating pathologists had been provided with the results/conclusions of our previous study as cited above, the cases were renumbered and the same 60 H&E stained slides were re-examined by the same three pathologists in conjunction with corresponding p16 and Ki67 immunostained slides prepared as previously described (Walts, Lechago, Bose, 2006a). The diagnoses were compared statistically for intra- and interobserver variability.

### Immunostains

Imunostaining for p16 and Ki67was performed in accordance with the manufacturers’ recommendations, as previously described (Walts, Lechago, Bose, 2006a). Nuclear and/or both nuclear and cytoplasmic staining in >10% of squamous cells was interpreted as positive for p16; cytoplasmic staining alone was considered nonspecific and interpreted as negative. For cases with condyloma and/or AIN, p16 was evaluated in the area(s) exhibiting the highest grade of atypia or dysplasia. The staining pattern for p16 was recorded as band-like when >90% of contiguous squamous cells stained positive. For cases exhibiting a band-like staining pattern, the location of the band was recorded as level 1, 2, or 3 (confined to the lower third, lower two-thirds, or full thickness, respectively of the squamous epithelium). Immunostaining for Ki67 was exclusively nuclear and recorded as positive when present in >50% of lesional squamous cells in the area corresponding to the diagnosis and the location of Ki67 positive cells was recorded as level 1–3.

### Statistical analysis

Results were compared statistically using weighted kappa analysis (StrataCorp, 2005. Stata Statistical Software: Release 9. College Station, TX: Stata-Corp LP).

## Results

### Intraobserver variation in diagnosis

As shown in Table 1, each of the three participant pathologists changed a substantial number (23%–48%) of their initial 60 diagnoses after reviewing the p16 and Ki67 immunostains. For each pathologist, the changes in diagnoses comprised a mix of increases (8%–20%) and decreases (3%–33%) in severity of diagnoses. Overall, a decrease in severity of diagnoses was more frequent. Of the total 63 diagnoses that were changed by the three participant pathologists, 54 (86%) were one-step and 9 (14%) were two-step changes in diagnosis. The two-step changes were equally distributed among the three participant pathologists. As shown in Table 2, addition of p16 and Ki67 immunostains improved concurrence with consensus diagnoses for two of the pathologists, while there was no net effect on the concurrence with consensus diagnoses for the third participant.

### Interobserver variation in diagnosis

**Figure 1.**
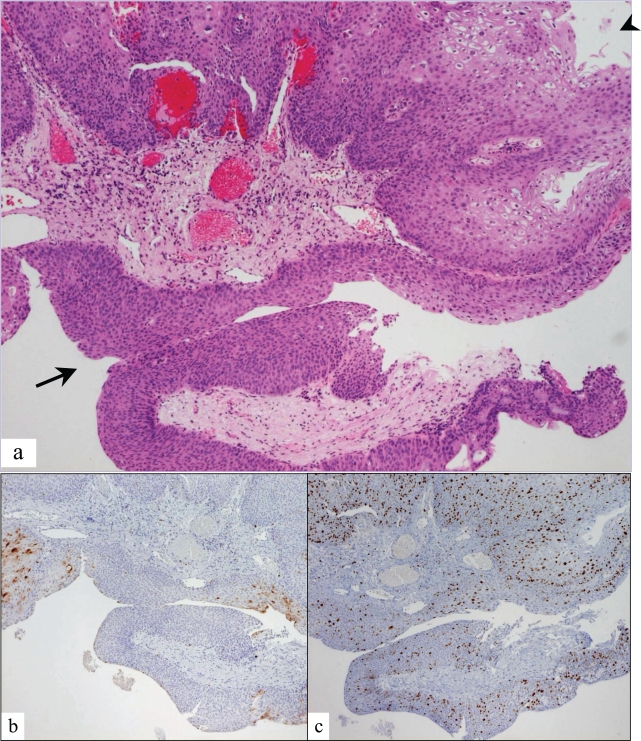
Anal biopsy showing **a**) condyloma (arrowhead) and squamous epithelium equivocal for high grade AIN (arrow); **b**) spotty p16 positivity in condyloma and absence of staining in epithelium equivocal for AIN; **c**) Ki67 positivity in <50% of nuclei.

**Figure 2.**
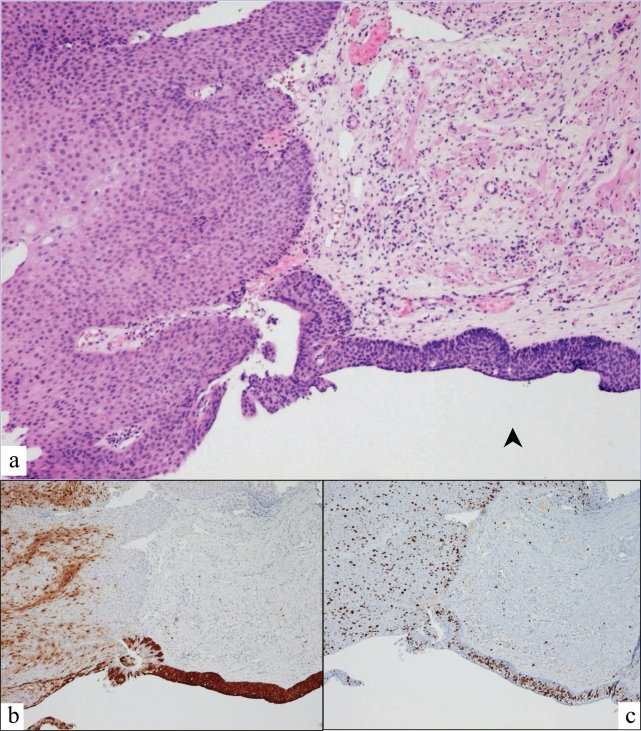
Anal biopsy showing **a**) atypical transitional epithelium equivocal for high grade AIN (arrowhead); **b**) band-like p16 positivity in high grade AIN and **c**) Ki67 positivity in >50% of nuclei in high grade AIN.

Table 3 compares the interobserver variation in diagnosis, while Table 4 compares the concurrence with consensus diagnoses before and after addition of p16 and Ki67 immunostains. Addition of p16 and Ki67 immunostains eliminated all five of the two-step differences and decreased the number of one-step differences in diagnosis among participating pathologists from 31 to 23 cases (26%), but yielded two cases for which there was no diagnostic agreement among the participants. Addition of p16 and Ki67 immunostains increased the percentage of cases in which all three participant pathologists agreed with each other by 18% and by which all agreed with the consensus diagnoses by 22% corresponding to an increase in overall multirater weighted kappa from 0.45 to 0.57. Figures 1 and 2 illustrate two cases in which stains for p16 and Ki67 reconciled disparate interpretations of anal biopsies. The anal biopsy in Figure 1 was interpreted as condyloma and high grade AIN on H&E evaluation. Spotty p16 positivity and Ki67 positivity in <50% of nuclei resulted in reinterpretation as condyloma without AIN. The anal biopsy in Figure 2 was interpreted as atypical transitional epithelium on H&E evaluation. Based on band-like p16 positivity and Ki67 positivity in >50% of nuclei, the interpretation was changed to high grade AIN. The presence of spotty p16 staining and Ki67 staining in <50% of nuclei in the adjacent thickened squamous epithelium is supportive of condyloma.

As shown in Table 5, addition of p16 and Ki67 immunostains resulted in substantial improvement in diagnosis agreement and in kappa values for negative and high grade AIN cases. Kappa values for low grade lesions were essentially unchanged.

## Discussion

AIN was first described as a potentially premalignant change by Fenger and Thue Nielsen (Fenger and Thue Nielsen, 1981) who reported histological changes in anal epithelium utilizing a three grade system (Fenger and Thue Nielsen, 1986) similar to that which had been developed by Richart (Richart, 1967) for cervical dysplasias. Acknowledging the subjectivity in the histologic assessment and grading of cervical dysplasia and the difficulties in patient management that intra- and interobserver variation in histologic diagnosis can create, Richart (Richart, 1990) subsequently proposed a modified two-tier system for grading cervical dysplasia. This division of squamous dysplasias into low grade and high grade intraepithelial lesions has now been widely adopted for the cervix as well as the anal canal by histopathologists, cytologists (Solomon et al. 2002) and clinicians.

The recent increase in AIN, in concert with the availability of cytology and anoscopy for diagnosis, development of a variety of treatment modalities for AIN and anticipation of vaccine(s) directed against HPV subtypes has renewed concerns about the impact of intra- and interobserver variation in diagnosis and grading of AIN on patient management (Carter et al. 1994; Colquhoun et al. 2003; Lytwyn et al. 2005). In a study of H&E stained sections from 100 anal biopsies with five reviewer pathologists using seven permitted diagnoses (normal, inflammation/HPV, AIN I, AIN II, AIN III, squamous carcinoma, inadequate), Carter et al. 1994 reported only moderate levels of agreement (weighted kappa scores ranging from 0.17 to 0.60) and expressed concern that the inability to reliably distinguish between grades of AIN may result in overtreatment of as many as two-thirds of patients. Similarly, in a study where four reviewer pathologists utilized three diagnoses (negative, low grade AIN, high grade AIN) to evaluate 155 anal biopsies, Lytwyn et al. 2005 reported an overall multirater weighted kappa score of 0.59. There was substantial agreement in the diagnosis of negative vs. high grade AIN (kappa scores 0.63 and 0.68, respectively) but only moderate agreement in the diagnosis of low grade AIN (kappa = 0.44). Colquohoun et al. 2003 also reported poor to only moderate agreement in diagnosis when three pathologists evaluated 190 anal biopsies utilizing six permitted diagnoses (normal, HPV change only, AIN I, AIN II, AIN III, invasive carcinoma). In their study, the weighted kappa scores achieved for HPV ranged from 0.24 to 0.53 while the scores for dysplasia ranged from 0.38 to 0.7. They reported agreement between the original pathologist and the three reviewing pathologists in only 32% of cases and noted that there was (a) the greatest level of disagreement for slides initially interpreted as AIN, (b) poor agreement for HPV changes, (c) disagreement in diagnoses at all levels of AIN and for the presence of invasive carcinoma. In contrast to the study by Carter et al. 1994 which only assessed interobserver variation in diagnosis, Colquohoun et al. 2003 also evaluated intraobserver variation in diagnosis for one pathologist reporting only moderate agreement except for invasive carcinoma where there was perfect intraobserver agreement for this individual. They suggested that intra- and interobserver variation in diagnosis could be decreased by utilizing (a) a two-tier (low grade, high grade) diagnosis system and (b) molecular biology markers rather than or in conjunction with routine histology.

We are aware of only one other study that addressed the effect of immunostaining on intra- and interobserver variation in the diagnosis and grading of anal biopsies for AIN. In an abstract, Haber et al. 2004 reported that immunostains for Ki67 were helpful in distinguishing dysplastic from nondysplastic anal biopsies. They utilized image analysis (CAS 200) to assess the number of Ki67 positive nuclei in the upper two thirds of the epithelium in 102 anal biopsies. Their study involved three participant pathologists using four permissible diagnoses (no dysplasia, mild, moderate, and severe dysplasia). They reported improvement in interobserver agreement and increase in kappa scores from 0.498–0.611 to 0.609–0.678. They concluded that immunostaining for Ki67 is helpful in reducing interobserver variability in grading AIN and state that Ki67 was “most helpful in discriminating between those cases with and without dysplasia”.

We assessed the potential value of immunostains for p16 and Ki67 interpreted in accordance with conclusions previously reached (Walts, Lechago, Bose, 2006a), to reduce intra- and interobserver variation in diagnosis of 60 anal biopsies reviewed by three pathologists. Briefly p16 is a cyclin-dependent kinase inhibitor and regulator of the transition from G1 to the S phase of the cell cycle that normally serves as a tumor suppressor. This gene product is upregulated and overexpressed immunohistochemically in most high risk HPV induced high grade cervical (Sano et al. 1998) and anal dysplasias and carcinomas. Hence, demonstration of overexpression of p16 coupled with a marked increase in Ki67 positivity should more closely reflect HPV induced AIN than Ki67 positivity alone. Our study confirms the existence of significant intra- and interobserver variation in assessment and grading of AIN when based only on routine stains. Our findings (illustrated in Figs. 1 and 2) indicate that when interpreted in accordance with our previously derived guidelines, immunostains for p16 and Ki67 are helpful in reducing variation in the diagnosis and grading of AIN. While addition of the immunostains improved the kappa values for each diagnostic category, the improvement was most significant for the negative and high grade AIN categories. Based on our present findings, we recommend that p16 and Ki67 immunostains be incorporated into the histopathologic evaluation of anal biopsies for HPV associated AIN. The benefit is most apparent in diagnostically difficult cases particularly those instances when the differential diagnosis includes high grade AIN as evidenced in our previous study of selected diagnostically difficult cases of AIN (Walts et al. 2006b). Addition of p16 and Ki67 immunostains in the evaluation of these cases can reduce false positive as well as false negative diagnoses of high grade AIN.

## Figures and Tables

**Table 1.** Intraobserver Variation in Diagnosis: H&E vs. H&E+p16 and Ki67 (N = number of cases Total = 60 cases).

	**Changed diagnoses**	**One-step change**	**Two-step change**	**Severity of diagnosis**	
				**Increased**	**Decreased**
MD “A”	20 (33%)	17	3 (15%)	5	15
MD “B”	14 (23%)	12	2 (14%)	12	2
MD “C”	29 (48%)	25	4 (16%)	9	20

**Table 2.** Intraobserver Variation in diagnosis: Agreement with consensus diagnoses (N = number of cases Total = 60 cases).

	**Diagnosis agrees with consensus diagnosis**	
	**H&E**	**H&E+p16 and Ki67**
MD “A”	40	49
MD “B”	51	51
MD “C”	32	41

**Table 3.** Interobserver Variation (N = number of cases Total = 60 cases).

	**H&E**	**H&E+p16 and Ki67**
All 3 participant MDs agree	24	35
Only 2 participant MDs agree	36	23
No 2 participant MDs agree	0	2
One step difference in diagnosis	31	23
Two step difference in diagnosis	5	0

**Table 4.** Interobserver Variation in diagnosis: Agreement with consensus diagnosis (N = number of cases Total = 60 cases).

	**H&E**	**H&E+p16 and Ki67**
All 3 participant MDs agree with consensus diagnosis	20	33
Multirater kappa	0.4486	0.5657
Total diagnoses that agreed with consensus diagnoses*	123 (68.3%)	141 (78.3%)

* Total diagnoses = 180 (60×3)

**Table 5.** Interobserver Variation: Cases with interobserver agreement in diagnosis (N = number of cases with all three participant pathologists agreeing on the diagnosis, Total = 60 cases).

**Diagnosis**	**H&E (kappa)**	**H&E+p16 and Ki67 (kappa)**
Negative	6 (0.42)	13 (0.59)
Low grade AIN	7 (0.32)	7 (0.35)
High grade AIN	11 (0.61)	15 (0.75)
Overall weighted kappa	0.45	0.57
